# ASO Author Reflections: Navigating Axillary Management for Residual Nodal Disease after Neoadjuvant Chemotherapy in Breast Cancer

**DOI:** 10.1245/s10434-026-20194-2

**Published:** 2026-07-20

**Authors:** Nicole Rademacher, Kristy K. Broman, Lily A. Gutnik

**Affiliations:** 1 Department of Surgery, University of Alabama at Birmingham, Birmingham, AL USA; 2 Institute for Cancer Outcomes and Survivorship, University of Alabama at Birmingham, Birmingham, AL USA; 3 The University of Texas MD Anderson Cancer Center, Houston, TX USA

## Past

Sentinel lymph node biopsy (SLNB) after neoadjuvant chemotherapy (NAC) may be used to restage the axilla. Prior work has demonstrated that for patients who convert to node-negative after NAC, axillary lymph node dissection (ALND) may be omitted.^1,2^ However, in patients with residual axillary disease, ALND remains the current NCCN standard of care.^1^

## Present

Ongoing clinical trials including Alliance A011202, TAXIS, and ADARNAT are evaluating axillary management strategies among patients with residual nodal disease after NAC. Understanding current real-world practice patterns in this population may help to identify gaps between current evidence and clinical practice. This study evaluated factors associated with omission of ALND after SLNB among patients with clinical node-positive breast cancer who had residual nodal disease following NAC.^3^ Factors associated with omission of ALND after SLNB, when adjusting for sociodemographic, geographic, and clinical variables, were lower pathologic nodal stage, partial mastectomy (compared to mastectomy), and omission of nodal radiation. In addition to these clinical factors, associations were also seen by Commission on Cancer facility type: compared with Academic Programs, patients treated at Comprehensive Community, Integrated Network, Community, and unknown program type had greater odds of omission of ALND after SLNB. Additionally, this study showed that across all facility types, ALND alone decreased while SLNB alone increased over the study period.

## Future

While this study found that omission of ALND was less common at Academic Programs, the reason behind this finding is unclear. Community Programs may treat older patients with more comorbidities and have a different payer mix, which could influence the results despite efforts to adjust for these factors in the analysis.

The increase in SLNB alone across facility types among patients with residual disease after NAC suggests that providers may be omitting ALND based on anticipation of ongoing clinical trial results. Real-world practice patterns seen in this study are aligned with other literature suggesting that omission of ALND in select patients with residual disease may be safe, particularly when radiation is planned.^4^ Axillary recurrence rates are low, and survival is not impacted by ALND for the majority of patients. A meta-analysis that evaluated nine studies found that ALND omission was not associated with a difference in overall survival or axillary recurrence at 5 years in patients with a positive SLNB after NAC.^5^ Additionally, ALND is associated with greater morbidity, namely lymphedema, than SLNB.

However, this study demonstrated that omission of radiation therapy was also associated with omission of ALND which deviates from existing standards and is not supported by current evidence in the literature. This raises concern that providers may be extrapolating evidence supporting SLNB alone without completion ALND or radiation therapy to populations in which it has not yet been thoroughly studied. This presents the opportunity to improve delivery of evidence-based care. Strategies to ensure guidelines are being appropriately applied should include systematic dissemination of new research findings and regular multi-disciplinary tumor board meetings.

As we await the results of Alliance A011202, TAXIS, and ADARNAT trials, surgical management of the axilla when there is residual disease after NAC should be based on the best available current evidence, multidisciplinary discussion, and patient-centered decision making.
